# Hydrogeochemical and environmental isotope study of Topusko thermal waters, Croatia

**DOI:** 10.1007/s10653-024-01904-9

**Published:** 2024-03-14

**Authors:** Mirja Pavić, Maja Briški, Marco Pola, Staša Borović

**Affiliations:** Croatian Geological Survey, Sachsova 2, 10000 Zagreb, Croatia

**Keywords:** Thermal water, Hydrochemical analyses, Stable water isotopes, Groundwater mean residence time, Topusko

## Abstract

Thermal waters in Topusko (Croatia), with temperatures of up to 65 °C, have been used for heating, health, and recreational tourism for the past fifty years. Hydrogeochemical monitoring can provide insights into deeper geological processes and indicate system changes from baseline levels. It helps to identify potential anthropogenic impacts, as well as natural changes. Hydrogeochemical, geothermometrical, and environmental isotope studies of thermal waters in Topusko were conducted to improve the existing conceptual model of the Topusko hydrothermal system (THS), providing a baseline for continuous monitoring of the thermal resource. 2-year thermal springs and precipitation monitoring took place from March 2021 until March 2023. Major anions and cations, stable and radioactive isotopes (i.e. ^18^O, ^2^H, SO_4_^2−^, ^3^H and ^14^C) and geothermometers were used to assess the origin of thermal waters in Topusko and their interaction with thermal aquifer. The results indicate the meteoric origin of thermal water, which was recharged in colder climatic conditions around the late Pleistocene–Early Holocene. Thermal water was last in contact with the atmosphere before approximately 9.5 kyr. Ca-HCO_3_ hydrochemical facies suggests carbonate dissolution as the dominant process driving the solute content. Geothermometrical results indicate an equilibrium temperature in the reservoir of 90 °C.

## Introduction

Thermal waters, characterised by elevated temperature values and unique chemical compositions, are valuable natural resources with applications in energetics, recreation, and therapy. Studying hydrogeochemical properties and environmental isotopes in thermal waters provides insights into their origin, hydrological processes, and water–rock interactions. These waters, ranging from 20 °C to above 225 °C globally, exhibit distinct geochemical characteristics influenced by geological, hydrogeological, and thermal characteristics. Comprehending their hydrogeochemical behaviour is essential for sustainable resource management, geothermal exploration, and environmental monitoring (Ármannsson & Fridriksson, 2009).

Examination of aqueous geochemistry is important in all phases of geothermal aquifer exploration, evaluation, and utilisation (Haizlip, 2016; Marini, 2004). Hydrogeochemical monitoring helps evaluate hydrothermal systems by determination of the geochemical baseline levels, tracking changes, and assessing the impact of water abstraction on the system. It involves analysing water chemistry, subsurface temperatures, and thermal and non-thermal groundwater interactions. Continuous geochemical surveys enable comparisons of existing and new data, aiding in the detection of anthropogenic impacts (i.e. the response of the aquifer to production stress) and natural variations (i.e. climate change and modifications of flow pathways due to earthquakes). Monitoring is essential for resource protection, legislative compliance, documentation of disturbances or natural changes, and scientific research (Heasler et al., 2009; Pryer, 2021). Continuous datasets are required to ensure adequate quantities of fluids with the necessary temperatures and pressures in the geothermal aquifer.

The chemical composition of groundwater is usually determined by the original composition of the infiltrated water and factors like altitude, vegetation, climate, aquifer mineralogy, and chemical reactions during its flow (Mazor, 2004). The analysis of the major ion and isotopic content, *in situ* parameters, spatial distribution, water composition evolution, and hydrochemical identification of water type are all useful tools for evaluating water chemistry and comprehensive characterisation of hydrothermal systems (Hounslow, 1995; Young, 1985). These analyses serve multiple purposes, including the differentiation of water types by observing the major ions content and determining parameters such as the temperatures attained at different depths (Verma et al., 2008) or the water mean residence time (MRT) (Plummer & Glynn, 2013). Moreover, they offer insights into the mineralogical composition of the aquifer and potential mixing with water from shallower cold aquifers in the spring areas (Blake et al., 2016). Various chemical geothermometers are used to estimate the aquifer equilibrium temperatures (Blasco et al., 2019; Karingithi, 1984). Tritium concentrations are used to assess the potential mixing of thermal waters with shallow groundwater (Janik et al., 1985; Lewis et al., 1989). Comparing stable water isotopes δ^18^O and δ^2^H in thermal water with the local meteoric water lines helps confirm/identify thermal water origin (Rman, 2016; Szocs et al., 2013). Analysis of ^14^C and δ^13^C of thermal water dissolved inorganic content is often used to estimate the time of thermal water infiltration into the subsurface in the assumed recharge area. Finally, analyses of δ^34^S and δ^18^O serve as a tool for assessing the origin of sulphates in the thermal water (Miljević et al., 2013; Porowski, 2014; Thiébaud et al., 2010).

The thermal springs in Topusko, situated in Central Croatia, reach temperatures of up to 53 °C. These springs are located in an area characterised by elevated heat flow at the southwest edge of the Pannonian Basin System (Horváth et al., 2015), representing a component of an intermediate-scale hydrothermal system. Geothermal systems exhibit distinct chemical compositions that influence their potential applications, and based on Moeck’s (2014) classification, Topusko is a non-magmatic conduction-dominated hydrothermal system (CD2d type). Despite limited prior investigations, thermal water in Topusko has been extensively utilised for district heating, health and spa purposes since the 1980s. Pavić et al. (2023) examined the historical geochemical data of thermal water, identified a possible fault zone responsible for thermal water outflow, and conducted a step-drawdown test to assess the transmissivity of the aquifer. Understanding the hydrogeochemical behaviour, thermal characteristics, and isotopic signatures of these waters is crucial for sustainable resource management, geothermal energy utilisation, and therapeutic applications.

Despite the prominence of Topusko as a thermal water site, comprehensive scientific investigations encompassing hydrogeochemical, geothermometric, and environmental isotopic aspects are limited. Therefore, this study aims to bridge this research gap by conducting an integrated analysis of the hydrogeochemical properties and environmental isotopes in Topusko thermal waters.

During the two-year research, the primary objective was to thoroughly monitor thermal springs and thermal water while establishing a geochemical baseline. Additionally, the study examined water–rock interactions, provided a detailed characterisation of thermal water geochemistry, determined aquifer equilibrium temperatures, investigated the sulphate origin in thermal water, and estimated the water MRT within the system.

Investigation of Topusko thermal springs adds to the growing body of knowledge about this particular location and offers information on the broader subject of geothermal research and utilisation. It emphasises the essential role that hydrogeochemical monitoring has in unravelling the complexities of hydrothermal systems, protecting these resources, and fulfilling the needs of scientific research and energy production.

## Materials and methods

### Study area: geological and hydrogeological setting

The thermal springs in Topusko are located in Central Croatia, within the southwest edge of the Pannonian Basin System (PBS). Pannonian part of Croatia is characterised by a higher than average geothermal gradient (49 ℃/km) and heat flux (76 mW/m^2^) due to back-arc crustal thinning in the PBS (Bošnjak, 1998; Horváth et al., 2015). These thermal waters have been used for centuries and have played a fundamental role in the development of tourism and healthcare facilities over the past five decades (Borović & Marković, 2015). The climate in the study area is moderate continental, slightly influenced by the Mediterranean climate of the northern Adriatic (Zaninović et al., 2008). The average annual precipitation is approximately 900 mm, and the annual average air temperature is 10.0 °C (DHMZ, 2021).

Thermal springs are generally part of intermediate-scale hydrothermal systems, including recharge areas in the mountainous hinterlands and geothermal aquifers, which are mainly hosted in Mesozoic carbonate rocks in Croatia (Borović et al., 2016). According to Šimunić (2008), the aquifer receives recharge from dolomite deposits outcropping west of Petrova Gora nappe. As a result, both the local and regional contexts must be considered.

**Fig. 1:**
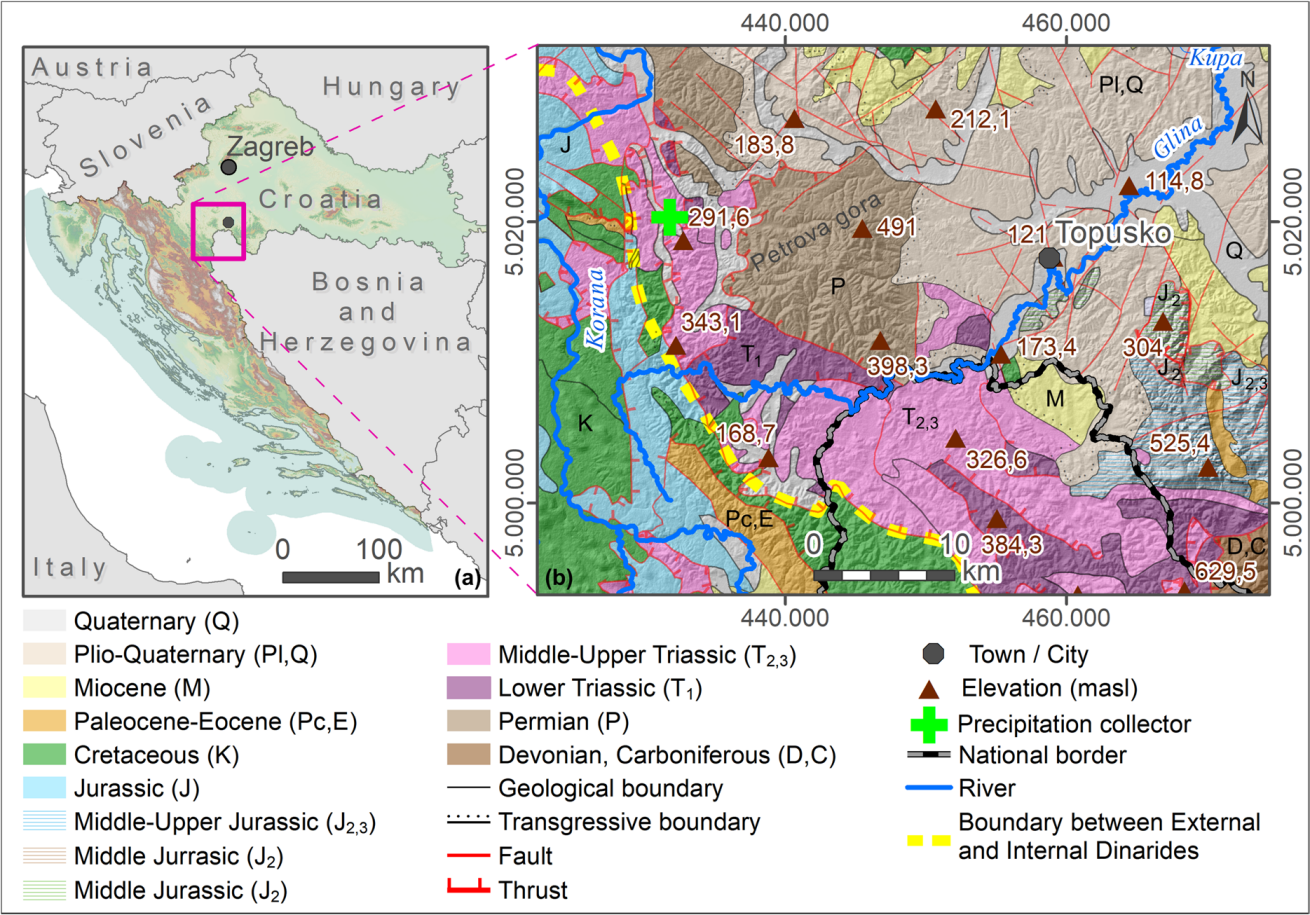
Geographical position (**a**) and geological map of a wider study area of THS (**b**) (HGI-CGS, 2009; Korolija et al., 1980a; Savezni geološki zavod, 1970)

The wider study area (W from Topusko) belongs to the Internal Dinarides tectonic unit and is situated at the NE margin of the Dinarides and SW margin of the PBS (Horváth et al., 2015; Pavelić & Kovačić, 2018; Schmid et al., 2004). In the west, the study area is bounded by a tectonic contact dividing Internal from External Dinarides (Fig. 1). The Internal Dinarides consist of a set of complex nappe sheets comprised of continental-derived material sedimented at the distal edge of the Adriatic microplate (Schmid et al., 2008). The External Dinarides are characterised by very thick sequences of Mesozoic carbonates, up to 8 km, deposited at the Adriatic Carbonate Platform (Schmid et al., 2004; Vlahović et al., 2005). Figure 1 shows that the majority of the THS study area is comprised of Late Paleozoic and Triassic deposits (P, T_1_, T_2_), while in the vicinity of the thermal water discharge area, the Holocene, Quaternary and Plio-Quaternary deposits (H; Q; Pl, Q) cover up older rocks, structures, and faults, which makes subsurface geological relations quite challenging to reconstruct.

According to the Basic geological map sheet Slunj (Korolija et al., 1980b), the oldest deposits in the research area belong to the clastic development of the younger Paleozoic found on Petrova Gora (P), schists, quartz-greywacke sandstones, shales, and fine-grained conglomerates of questionable total thickness. The Lower Triassic deposits (T_1_) continuously follow clastic development, composed of red-violet mica-schist, light reddish mica-schist sandstones, and grey-greenish schist marls. The Middle and Upper Triassic deposits (T_2,3_) consist predominantly of carbonate limestone and dolomite rocks, which are intensively karstified south of the Topusko. SW from the thermal spring area, Jurassic sedimentary, metamorphic and orthometamorphic rocks (J_2,3_; J_2_) outcrop to small extents, belonging to the ophiolitic-sedimentary thrust complex. They are represented by quartz-greywacke sandstones, shales and cherts, metamorphosed sediments (pelites and psammites), cherts, limestones and pyroclastic rocks, and amphibolites and amphibolite schist, respectively (Šikić et al., 2009). Deposits belonging to External Dinarides are dominantly represented by intensively karstified Mesozoic limestones and dolomites (J, K). Palaeocene (Pc, E) clastic deposits are also characterised by flysch development (conglomerates, sandstones, silt, marls, clays).

**Fig. 2:**
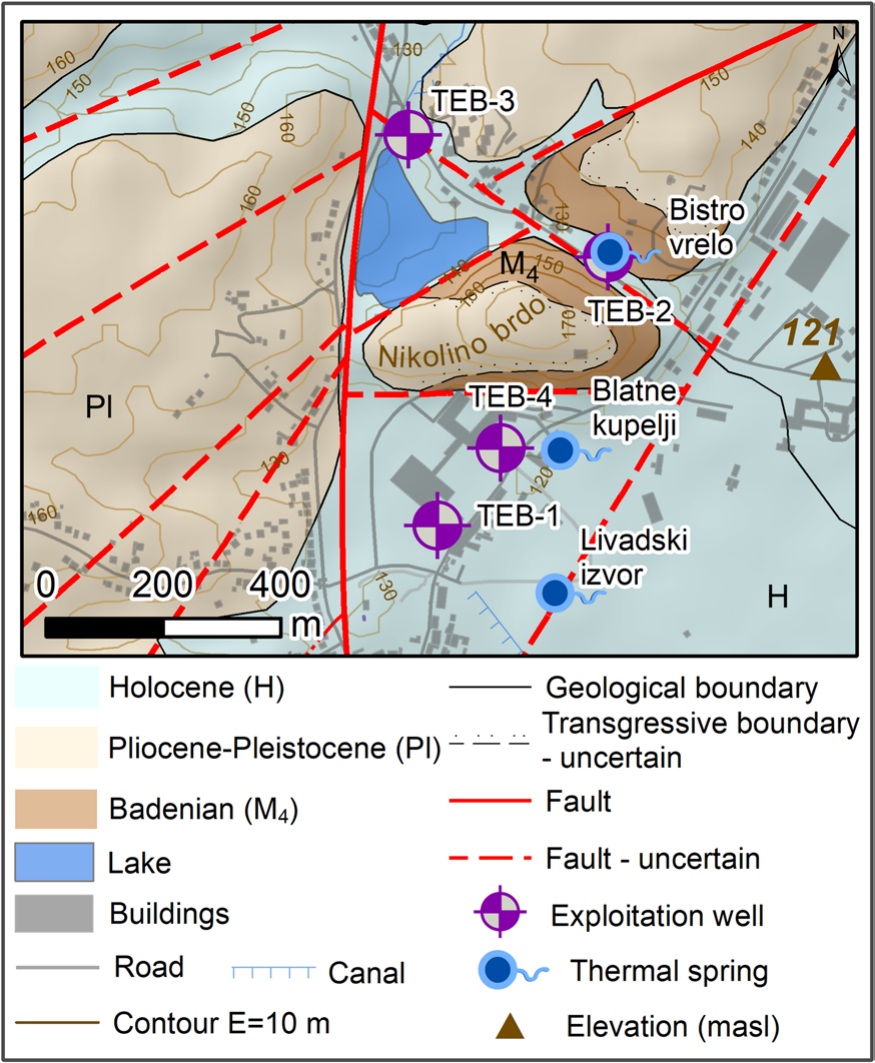
Geological map of the thermal spring area in Topusko. Locations of exploitation wells and natural thermal springs are presented: Blatne kupelji spring, Bistro vrelo spring, and Livadski izvor spring (modified after Pavić et al., 2023)

Neogene deposits (Figs. 1 and 2; M; M_4_; Pl,Q; Q (Pl. H)) are primarily transgressive to all older rocks. These sediments are represented by the surface occurrence of clastites, fine-grained and coarse-grained conglomerates, sandstones, silts, marls, clays, lithothamnium limestone, fine- to coarse-grained gravels, sands, and conglomeratic sands (Hrvatski geološki institut, 2009; Korolija et al., 1980b).

From a hydrogeological point of view, Triassic carbonates represent artesian geothermal aquifers in the area of the Topusko HTS. The complex of Paleozoic deposits, out of which Petrova Gora is mainly built, forms an impermeable core complex, together with individual lower Triassic elements of the structural setting (Bahun & Raljević, 1969; Šimunić et al., 2008). Low permeability younger Neogene deposits cover the geothermal aquifer in the discharge area.

In the area of Topusko, there are three natural thermal springs with a total capacity of approximately 25 L/s and temperatures ranging from 46 °C (Blatne kupelji) to 53 °C (Livadski izvor). There are four exploitation wells near the natural thermal springs. Exploitation wells TEB-1, TEB-2, TEB-3, and TEB-4 were drilled in the period 1982–1989. TEB-1 (243 m), TEB-3 (163 m), and TEB-4 (80.8 m) are used for spa and heating purposes, while TEB-2 is damaged and no longer in operation. Total well capacity is estimated at 200 L/s with water temperature of up to 65 °C. The wells are artesian with a pressure of 0.5 to 2.3 bar (Čubranić, 1984; Šegotić & Šmit, 2007). According to Šimunić et al. (2008), the spring area is bounded by three faults that form a block in the form of a three-sided prism, enabling the uplifting of Triassic carbonates, which was determined by drilling (Fig. 2). Pavić et al. (2023) identified fault damage zones in the spring area that provide a preferential pathway for groundwater upwelling to the surface from a confined geothermal aquifer.

### Water sampling and analyses

The monitoring of thermal waters and cumulative precipitation collection in the Topusko study area was conducted for two years, from March 2021 to February 2023 and April 2021 to March 2023, respectively. Sampling and monitoring points include two natural thermal springs, Livadski izvor and Blatne kupelji, and well TEB-4. The temperature was continuously monitored at the thermal springs using automatic data loggers (Onset HOBO Water level loggers 30, which were replaced by the Onset HOBO U12-015 Stainless Steel Temperature Data Logger in July 2021 due to repeated malfunctions caused probably by its exposure to higher water temperatures). Periodic monitoring included monthly *in situ* measurements, data retrievals from automatic loggers, and thermal water and cumulative precipitation sampling for subsequent hydrogeochemical analyses. After each sampling campaign, principal anions and cations, silica (SiO_2_) concentration, and stable water isotope content (^2^H and ^18^O) were analysed. In addition, water sampling was conducted during minimum and maximum water abstraction periods for radioactive isotope analyses (^3^H and ^14^C) and stable isotope analyses from sulphate anion (^34^S and ^18^O).

The monthly thermal water samples were collected in polyethylene bottles (Ármannsson, 2012) of 100- and 200-mL volume and stored at 4 °C until the upcoming analyses. Cumulative precipitation samples were acquired by Palmex RAIN SAMPLER RS1B, specially designed for collecting and storing samples without evaporation. *In situ* measurements of key physico-chemical parameters (temperature (T), pH and electrical conductivity (EC)) were conducted using the WTW Multi 3320 multiparameter probe. Additionally, alkalinity (bicarbonate concentration) was determined in the field using a digital titrator (HACH 16900), by volumetric, titrimetric method with 1.6 N H_2_SO_4_, and bromocresol green indicator.

Thermal water samples have been analysed at the Croatian Geological Institute laboratory for the major ions content using ion chromatography (Thermo Scientific Dionex ICS-6000 HPIC System). The analysis of stable oxygen and hydrogen isotopes in water was done using a Picarro analyser (Picarro L2130-i Isotope and Gas Concentration Analyser). During sample measurements, international standards produced by the USGS (isotope reference material USGS 46, USGS47, and USGS48) were also measured, allowing subsequent result calibration for each measurement. Measurement precision was ± 0.2 ‰ for δ^18^Ο and ± 1 ‰ for δ^2^H. Results are presented in delta notation (‰), normalised to the international measurement standard VSMOW (Vienna Standard Mean Ocean Water) (Craig, 1961; Mazor, 2004). Hach DR3900 Spectrophotometer was used to determine SiO_2_ content in the thermal water samples. Laboratory for low-level radioactivity of Ruđer Bošković Institute determined tritium activity concentration in two thermal water samples from well TEB-4 with the method of electrolytic enrichment (*LNA-PS 7.2/3 Determination of *^*3*^*H activity concentration)* using the liquid scintillation counter Quantulus 1220. The same laboratory determined relative specific ^14^C activity in the same samples by accelerator mass spectrometry (AMS) technique (AMS-14C) (Krajcar Bronić et al., 2010; Sironić et al., 2013). In addition, three thermal water samples were analysed in the Hydrosiotop laboratory in Germany to determine δ^34^S and δ^18^O values of sulphate anion. The isotopic compositions are given in traditional delta notations (‰) with respect to the VSMOW standard for oxygen and CDT (Canyon Diablo Troilite) for sulphur.

### Data processing methods

The acquired major ions data were processed in Excel and "Diagrammes V6.72" software (Simler, 2012). They were used to calculate total dissolved solids (TDS) content and saturation indexes of calcite and dolomite (SI) to evaluate chemical equilibrium in thermal water samples. The quality of the major ions analyses was tested by assessing the charge balance and its error through the equation:

1 $${\text{Charge}}\;{\text{ balance}}\;{\text{ error }}\left( {\text{\% }} \right){ = }\frac{{\sum_{{\text{cations }}} { - }\sum_{{{\text{anions}}}} }}{{\sum_{{{\text{ions}}}} }}{{ \times 100 (\% ) }}$$

where the ionic concentrations are in meq/L. Samples with a charge balance error of more than 5% were excluded from further analyses (Appelo & Postma, 2005; Mazor, 2004). The remaining dataset, together with measured in situ parameters, is presented graphically by box plot diagram and by descriptive statistic: arithmetic mean (Average), minimum (Min) and maximum (Max), and the standard deviation (St. dev).

Piper diagram (Piper, 1944) and molar and equivalent ratios of major ions (Ca^2+^ + Mg^2+^ vs HCO_3_^−^ + SO_4_^2−^, Ca^2+^ vs SO_4_^2−^, Na^+^ vs Cl^−^, etc.) were used to identify the hydrochemical facies of thermal water, the water–rock interaction processes, and the dominant lithology in the recharge area (Komatsu et al., 2021; Serianz et al., 2020; Xu et al., 2019).

The saturation indexes (SI) of the main minerals in the aquifer were calculated to assess whether these minerals are close to or far from equilibrium with their solubility products (Clark, 2015; López-Chicano et al., 2001). The index gives the saturation degree of the groundwater sample with respect to minerals based on the equation (Appelo & Postma, 2005):

2 $${\text{SI}} = \log \left( {\frac{{{\text{IAP}}}}{K}} \right),$$

where IAP is the ion activity product, and K represents the solubility product. The equilibrium conditions between the mineral and the solution are represented by a straight line on a logarithmic plot, where SI takes the zero value. If greater than zero, the mineral is supersaturated and can precipitate (SI > 0), and less than zero reflects undersaturation and possible dissolution if the specific mineral is present. According to many authors (Chelnokov et al., 2022; López-Chicano et al., 2001; Plummer et al., 1990; Serianz et al., 2020), the assumed range of SI uncertainty for calcite is ± 0.1 and ± 0.5 for dolomite. The accepted equilibrium range is indicated as a grey rectangle area in the results section. The study of the saturation index (SI) is crucial in assessing the potential for precipitation or dissolution of minerals in thermal waters. It also provides information on the water's capacity to corrode materials or deposit mineral scales, which is critical to understanding the impacts on subsurface infrastructure and environmental systems (Appelo & Postma, 2005; Taghavi et al., 2019).

Comparing the stable water isotopic composition (δ^2^H and δ^18^O) of precipitation and thermal spring water provides insight into the origin, residence time, and features of water transport through the system (Edwards et al., 2007; IAEA, 1970; Tijani et al., 2022). Excel and online statistic calculator Statistic Kingdom (2017) were used for stable water isotope data preparation, observation of the relationship between δ^2^H and δ^18^Ο, statistical analyses, determination of outliers and testing isotope content distribution for normality before construction of local meteoric water line (LMWL). The δ^2^H excess (d-excess; Dansgaard, 1964) was calculated for each sample following the equation:

3 $$d - {\text{excess }}\left( {{\permille}} \right) = {{ \updelta }}^{2} {\text{H}} - 8{\updelta }^{18} {\text{O}}$$

It can be interpreted as an index of deviation from the global meteoric water line GMWL (Craig, 1961), which has a *d*-excess value of 10‰. This excess, caused by kinetic evaporation (non-equilibrium) during the formation of the primary vapour mass, can be a valuable tool to determine the origin of water and conditions during the vapour formation (Clark, 2015). Linear regression model of precipitation stable water isotope data and Chauvenet's Criterion test (Taylor, 1997) on d-excess values were used to identify outliers before LMWL calculation, following the method described by Benjamin et al. (2005). A Quantile–Quantile plot (Q-Q plot) was used as a graphical tool, together with the Shapiro–Wilk W-test, to identify deviations from the normality of the data. LMWL was calculated using the ordinary least square regression (OLSR), excluding outlier data. This simple linear regression model is one of the three types of linear regression analyses recommended by the IAEA (Hughes & Crawford, 2012; IAEA, 1992). Finally, δ^18^O and δ^2^H values of thermal water samples were compared to OLSR LMWL to study the relationship between precipitation and groundwater.

Tritium can be used to determine the mean groundwater residence time or mixing processes in the aquifer since the concentration in groundwater reflects the atmospheric concentration when the water was last in contact with the atmosphere. The half-life of tritium is 12.32 years, and its concentrations are measured in tritium units (TU) (1 TU = 0.118 Bq l^−1^, which represents one ^3^H atom in 10^18^ atoms of hydrogen) (Fetter, 2001; IAEA, 2005, 2013; Rozanski et al., 1991). The classification after Motzer (2007) was used in this study: tritium content < 0.8 TU indicates the recharge at least before 1950, tritium activity concentrations of 0.8 − 4 TU suggest a mix of sub-modern and modern water, while 5 − 15 TU concentrations indicate modern recharge (< 5 to 10 years). Detection of tritium in thermal water could imply mixing with the groundwater of modern recharge, which can further be a sign of thermal water overexploitation.

NetpathXL software (Parkhurst & Charlton, 2008; Plummer et al., 1994) was used to correct the initial ^14^C activity (^14^C_0_) and to estimate radiocarbon ages of dissolved inorganic carbon (DIC) in a single thermal water sample, in which the initial and final water are defined as the same sample (Plummer & Glynn, 2013). This approach to radiocarbon dating is done without consideration of the geochemical mass balance reactions. Han and Plummer's graphical method (2012) was used to evaluate dominant geochemical processes occurring in geothermal aquifers, which affected the DIC carbon isotope content before ^14^C radioactive decay, and to qualitatively estimate the radiocarbon age of thermal water samples. The radiocarbon DIC groundwater age (t) in years BP can be estimated by applying the ^14^C decay equation, assuming advective piston-flow conditions:

4 $$t = - \frac{{t_{{1{/}2}} }}{\ln 2}\ln \left( {\frac{{{\phantom{i}}^{14} {\text{C}}}}{{{\phantom{i}}^{14} {\text{C}}_{0} }}} \right),$$

where *t*_1/2_ is the ^14^C half-life (Libby—5570 yr or physical 5730 yr), ^14^C content of DIC measured from the collected groundwater sample, and the initial ^14^C_0_ DIC value without considering impacts of geochemical processes on water chemistry (Geyh, 2000, 2005; IAEA, 1970). Radiocarbon dating of groundwater is undoubtedly one of the most challenging and frequently disputed applications of radiocarbon dating introduced by Münnich (1957) and Münnich and Roether (1967). Considerable challenges in the interpretation of presented results arise due to the potential influence of geochemical reactions ("reservoir effect"), such as carbonate dissolution, ion exchange, and isotopic exchanges, which can complicate the accurate ^14^C ages by altering the initial ^14^C content independently of radioactive decay. Geochemical processes often reduce the ^14^C content beyond radioactive decay, leading to apparently older than expected groundwater ages. In this work, for the application of traditional adjustment models, we use the lowercase 'pmc' for the ^14^C content of DIC (^14^C DIC), which represents the ^14^C content without normalisation (IAEA, 2013).

Different chemical geothermometers (silica and cation) were used to estimate the equilibrium temperature of thermal water in the aquifer. Chemical geothermometry represents the classical approach for estimating thermal water temperature within a deep aquifer. It relies on various empirical or experimentally derived calibrations based on temperature-dependent heterogeneous chemical reactions (Flóvenz et al., 2012). Classical chemical geothermometers use elemental content controlled by these reactions to infer the reservoir temperature (e.g. as seen in studies by Truesdell, 1976; Marini, 2004; Blasco et al., 2018). This approach assumes that these elemental contents remain unaltered during the water's ascent to the surface without significant modifications due to interactions with the surrounding rocks, attaining the equilibrium state.

Stable isotopes (δ^18^O and δ^34^S) of sulphate anion (SO_4_^2−^) in thermal water were compared with the graphical classification provided by Porowski (2014, 2019) to determine the origin of sulphates in thermal water, as successfully applied by many authors (Bouaicha et al., 2019; Eastoe et al., 2022; Fórizs et al., 2019; Miljević et al., 2013).

## Results and discussion

### Major ions chemistry

A total of 72 thermal water samples were analysed. Based on the calculated charge balance error, two samples collected from Livadski izvor and Blatne kupelji thermal springs were excluded from the analysis. Table 1 shows the mean values and ranges of the groundwater physico-chemical parameters measured in situ, the major ions, and the silica concentration.

**Table 1:** Descriptive statistics of in situ physico-chemical parameters, major ions, and silica content of Topusko thermal water

Sampling site	Statistics	T	pH	EC	TDS*	Ca^2+^	Mg^2+^	Na^+^	K^+^	HCO_3_^−^	SO_4_^2−^	Cl^−^	NO_3_^−^	SiO_2_
		°C	–	μS/cm		mg/L								
Livadski izvor spring	Mean	52.4	6.50	620	546	80.8	16.3	17.7	11.0	244.7	98.3	17.5	0.6	38.1
	Min	51.4	6.38	582	497	78.2	14.6	17.3	10.7	231.8	78.6	14.5	0.3	35.3
	Max	53.2	6.76	635	562	82.4	16.9	17.9	11.3	257.4	106.8	19.4	2.9	40.5
	St. dev	0.5	0.09	10	14	0.8	0.5	0.2	0.2	6.8	7.3	1.3	0.8	1.4
Blatne kupelji spring	Mean	48.0	6.85	636	556	83.4	16.7	17.9	11.1	249.6	100.7	17.87	0.5	37.8
	Min	44.1	6.51	593	503	78.7	14.8	17.6	10.9	233.0	81.6	14.64	0.3	28.6
	Max	51.9	7.40	650	574	86.0	17.3	18.2	11.5	258.6	108.7	19.68	0.6	41.0
	St. dev	2.3	0.31	13	15	1.8	0.5	0.2	0.2	6.0	7.4	1.3	0.1	2.3
Well TEB-4	Mean	64.1	6.56	626	555	82.0	16.7	17.9	11.2	248.9	100.0	17.63	–	39.1
	Min	61.1	6.35	607	502	79.6	15.0	17.5	10.9	234.2	75.4	12.81	–	36.3
	Max	65.2	6.71	670	577	82.8	17.2	18.2	11.5	268.4	109.2	19.60	–	41.4
	St. dev	1.0	0.09	14	17	0.7	0.4	0.2	0.2	7.7	8.7	1.7	–	1.5

Charge balance errors are ± 5%. *TDS was calculated using Diagrammes V6.72 software (Simler, 2012)

**Fig. 3:**
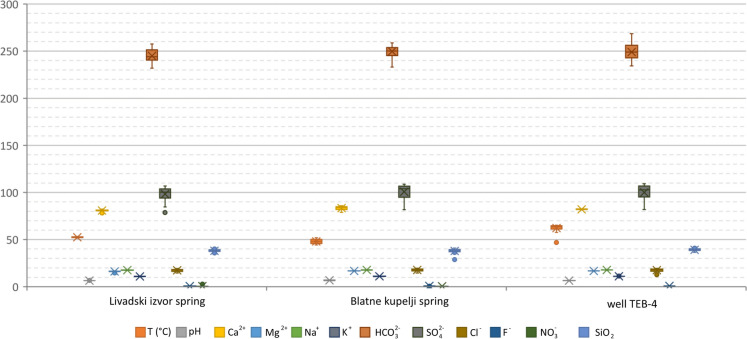
Box-plot of Topusko thermal water major ions (mg/L), temperature T (°C), and pH

The graphical representation of the data summarised in Table 1 is shown in Fig. 3 in the form of a box-plot diagram, which indicates that analysed thermal water samples originate from the same thermal aquifer and display constant properties over the monitored period.

Thermal water pH is slightly acidic, with average values in the monitored objects from 6.5 to 6.8. The electrical conductivity (EC) ranges from 582 μS/cm to 680 μS/cm, being increased by the temperature effect, where the parameter values are proportionally increased with respect to temperature and not due to high mineralisation or high concentrations of HCO_3_^−^ and SO_4_^2−^ (Hermans et al., 2014). Total dissolved solids (TDS) in thermal water range from 497 to 577 mg/L and serve as a good indicator of water mineralisation (Hiscock & Bense, 2014), which can be characterised as medium to low. The TDS values are within the range for thermal waters of Internal Dinarides, which generally show TDS lower than 1 g/L (Milenić et al., 2012). The generally low mineralisation of the thermal water indicates a precipitation recharge-dominated groundwater system, and water with a TDS < 1000 mg/L is considered fresh (Halle, 2004).

**Fig. 4:**
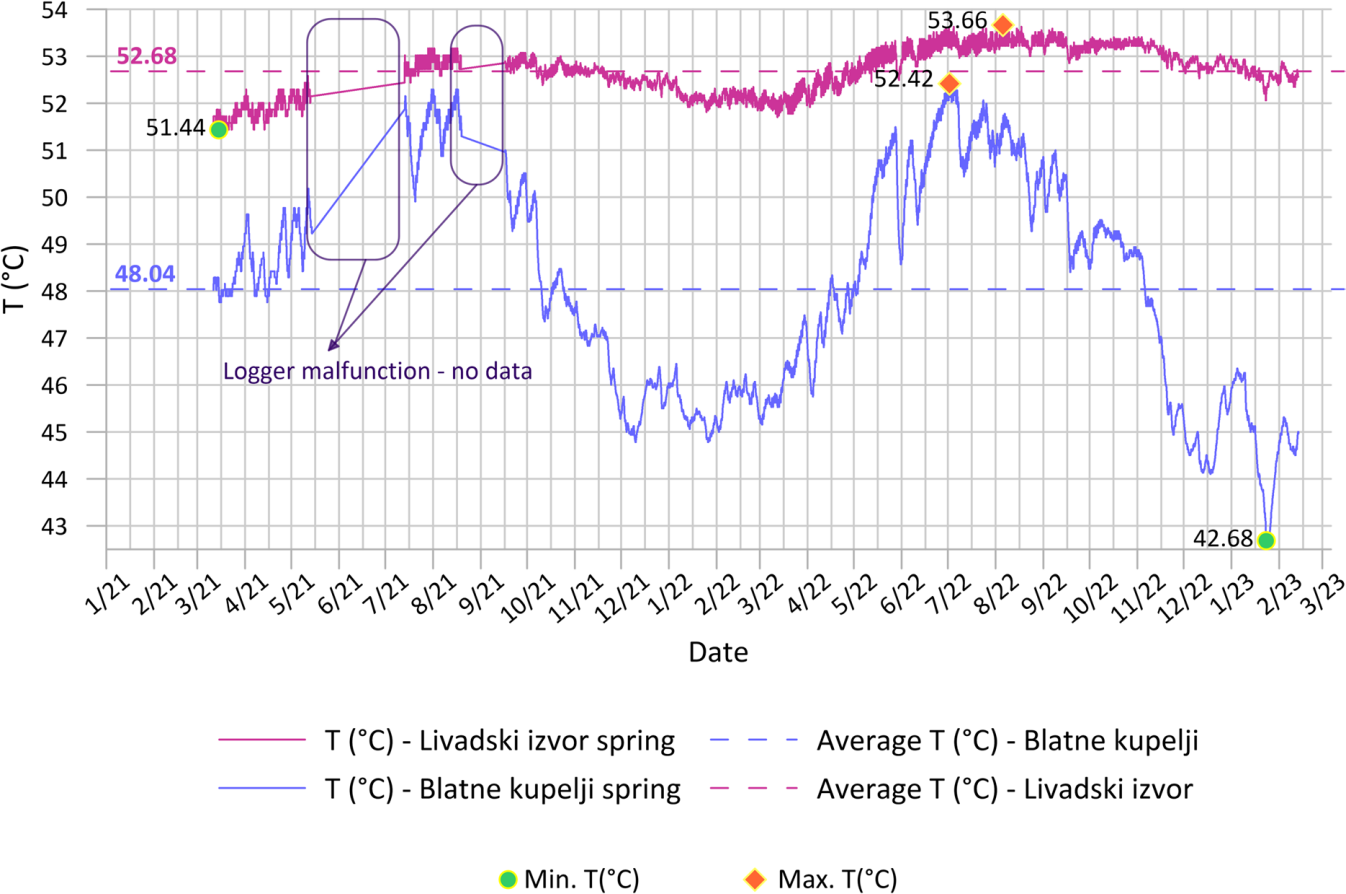
Continuous temperature data measured at thermal springs Livadski izvor and Blatne kupelji from March 2021 to February 2023

Continuous temperature measurements in thermal springs are presented in Fig. 4. The average recorded temperatures for thermal spring Livadski izvor are 52.68 °C and 48.04 °C for Blatne kupelji. Maximal measured temperatures are 53.66 °C and 52.42 °C, respectively. The temperature of thermal waters varies from 42.68 °C to 53.66 °C in the springs, while the temperature of water in TEB-4 well is 65 °C. Annual changes in temperature follow the seasonal changes in the air temperature, with more amplified amplitudes recorded at Blatne kupelji spring (up to 10 °C).

**Fig. 5:**
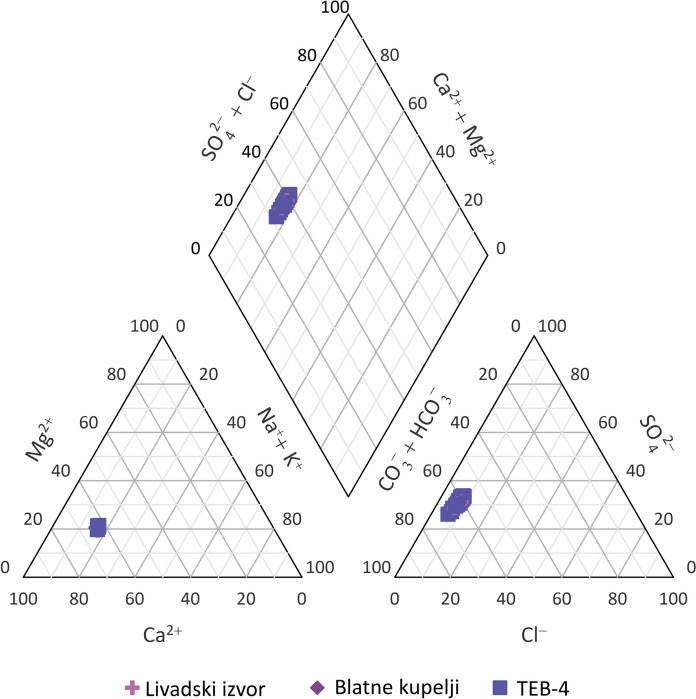
Piper diagram of thermal water samples from the discharge area of Topusko HTS (March 2021–February 2023)

In samples from Topusko thermal water, major cations concentrations follow the order Ca^2+^ > Na^+^ > Mg^2+^ > K^+^, with the dominance of Ca^2+^ (~ 80 mg/L) and comparable Na^+^ and Mg^2+^ concentrations (17 mg/L). The dominant anion in thermal water samples is HCO_3_^−^, ranging from 232 to 268 mg/L for all three sampling locations, followed by relatively high concentrations of SO_4_^2−^ anion, ranging from 75 to 109 mg/L. The composition of the major ions of thermal water is shown graphically using Piper's diagram (Fig. 5) (Piper, 1944). According to the composition of the major anions and cations, the samples show Ca-HCO_3_ hydrochemical facies (Freeze & Cherry, 1979), as indicated by the dominant presence of Ca^2+^ and HCO_3_^−^ in the Piper diagram. This composition suggests that the limestone is the dominant source of dissolved solutes in the aquifer and prevailing in the catchment area, together with dolomites, as the dominance of Ca^2+^ cation followed by Mg^2+^, with lower content of alkali metals, is characteristic of groundwater in worldwide carbonate aquifers (Goldscheider et al., 2010; Lei et al., 2022; Li et al., 2020; Patekar et al., 2022; Wang et al., 2020). Plotting of sample composition in the Piper diagram in almost the same spot indicates a large and stable system where the ion composition is more or less constant with no significant changes over time.

**Fig. 6:**
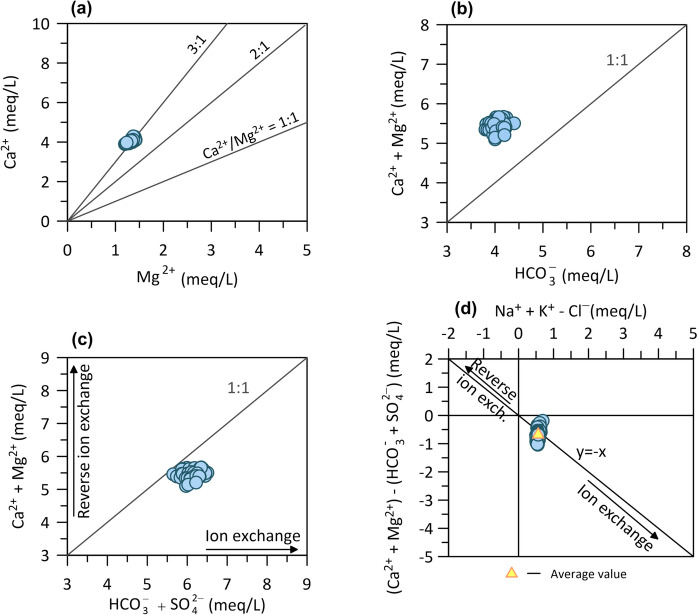
Biplots of **a** Ca^2+^ vs Mg^2+^, **b** (Ca^2+^ + Mg^2+^) versus HCO_3_^−^, **c** (Ca^2+^ + Mg^2+^) versus (HCO_3_^−^ + SO_4_^2−^) and **d** (Ca^2+^ + Mg^2+^) − (HCO_3_^−^ + SO_4_^2−^) versus Na^+^ + K^+^-Cl^−^

Assuming that the initial composition of Topusko thermal waters originates from precipitation, which is usually the primary recharge source for most groundwaters (Ármannsson, 2012), during the water cycle, the initial composition of water is altered mainly through rock-weathering, evaporation and other geochemical processes that occur in the aquifer (i.e. dissolution, ion exchange, sulphate reduction, etc.) (Appelo & Postma, 2005; Fetter, 2001; Halle, 2004; Mazor, 2004). In order to get a better understanding of water–rock interaction and geochemical processes governing the solute content in thermal water, biplot diagrams of molar or equivalent ratios of major anions and cations were studied (Clark, 2015; Garrels, 1976; Hounslow, 1995; Rman, 2016; Xu et al., 2019; Zhang et al., 2016). Due to the plausible flow of Topusko water in a carbonate aquifer, the Ca^2+^/Mg^2+^ molar ratio was investigated (Fig. 6a). The stoichiometry ratio of dominant dolomite dissolution would be 1 and mixed limestone with dolomite 2 (Fellehner, 2004; Gao et al., 2017; Hilberg & Schneider, 2011). Topusko water shows the equivalent ratio of 3, pointing to a surplus of Ca^2+^ over Mg^2+^. Such ratio suggests a prevailing interaction of thermal water with limestone, followed by dolomite, as well as possible additional sources of Ca^2+^.

Additionally, the relationship between (Ca^2+^  + Mg^2+^) and HCO_3_^−^ in Fig. 6b shows the surplus of cations over HCO_3_^−^, suggesting an additional source of Ca^2+^ besides carbonate dissolution. Possible sources could be ion exchange, gypsum dissolution, silicate weathering, or incongruent dissolutions of dolomite. The following bivariate plots were examined to narrow the occurrence of one or more of these processes. The scatter plot (Ca^2+^ + Mg^2+^) versus (HCO_3_^−^  + SO_4_^2−^) is commonly used to identify ion exchange processes (Fisher & Mullican, 1997; Nematollahi et al., 2016; Tay et al., 2015; Tziritis et al., 2016). As depicted in Fig. 6c, SO_4_^2−^ participates in balancing the solution in addition to HCO_3_^−^. The equiline represents the stoichiometry correlation between these ions, which assumes carbonate and gypsum (and anhydrite) dissolution as the dominant and equally represented process controlling solution composition. Samples show a ratio of 0.9 resulting from a (Ca^2+^ + Mg^2+^) depletion with respect to HCO_3_^−^  + SO_4_^2−^, suggesting the cation exchange process occurs in the aquifer along with carbonate minerals dissolution. As a result, Na^+^ and K^+^ must balance the excess in the solution's negative charges.

For further evaluation of the previously assumed ion exchange process, a bivariate plot (Ca^2+^  + Mg^2+^) − (HCO_3_^−^  + SO_4_^2−^) versus (Na^+^  + K^+^  − Cl^−^) was examined (Fig. 6d) (García et al., 2001; Xiao et al., 2017). Samples plotting at the centre of the plot would indicate the absence of ion exchange or reverse or ion exchange processes. In the case of ion exchange as a significant controlling process, the samples would plot on the line with slope − 1 (y =  − x). Results indicate that Na^+^, K^+^, Ca^2+^, and Mg^2+^ in Topusko thermal aquifer participate in ion exchange reactions. The graph in Fig. 6d shows the decrease in Ca^2+^ and Mg^2+^ content versus the increase in Na^+^ and K^+^. Such phenomena can be explained by an ion exchange process where Na^+^ is removed from clay minerals and replaced by Ca^2+^ from the solution:

$${\text{Ca}}^{{{2} + }} + {\text{2Na}} - {\text{Clay}} \to {\text{2Na}}^{ + } + {\text{Ca}} - {\text{Clay}},$$

The ion exchange process reduces the concentrations of Ca^2+^ and Mg^2+^ and increases the Na^+^ concentration in groundwater. Furthermore, the weathering of albite could also contribute to Na^+.^ According to Zhang et al. (2016), such a process might reflect longer groundwater residence times and longer flow paths, facilitating cation exchange reactions between the thermal water and aquifer matrix. Marković et al. (2015) showed an additional process of ion exchange occurring along carbonate dissolution and controlling the major ion chemistry in the thermal waters of Hrvatsko Zagorje.

**Fig. 7:**
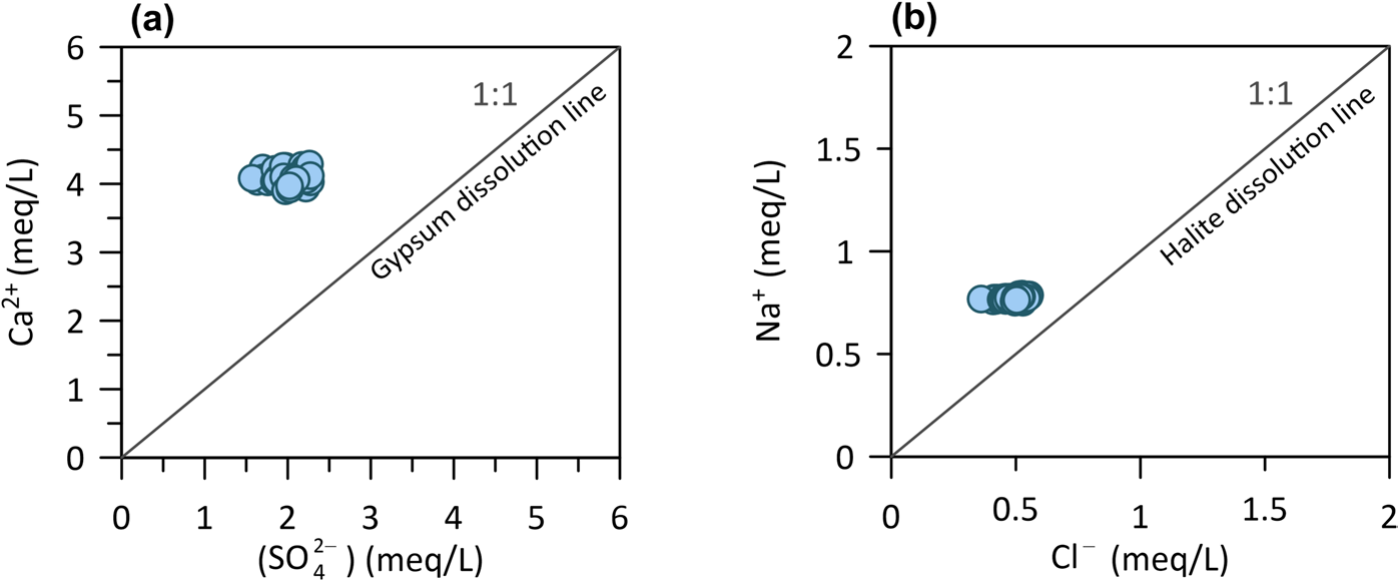
Biplot scatter diagrams of **a** Ca^2+^ versus SO_4_^2−^ and **b** Na^+^ vs Cl^−^

The occurrence of evaporite mineral gypsum dissolution in the thermal aquifer was investigated through the Ca^2+^/SO_4_^2−^ (Fig. 7a). Usually, the primary assumption regarding sources of sulphates in the groundwater is the dissolution of gypsum and/or anhydrite, which results in the Ca^2+^/SO_4_^2−^ stoichiometry equivalent ratio of 1. Topusko water has an average value of the Ca^2+^/SO_4_^2−^ ratio 2, which distributes all samples above the gypsum dissolution line. An excess of Ca^2+^ relative to sulphate anion indicates other sources of cation in addition to gypsum, such as calcite, dolomite or silicates (i.e. minerals like feldspar). Pavić et al. (2023) investigated historical chemical analyses of Topusko water and argued that gypsum dissolution is a minor process together with the dominant carbonate dissolution.

Dissolution of halite was additionally investigated to evaluate the source of Na^+^ (Fig. 7b). Na^+^ vs Cl^−^ biplot shows most samples plot above 1:1 equiline, with the ratio value of 1.5, suggesting that halite dissolution is not a primary source of Na^+^. Excess of Na^+^ over Cl^−^ indicates other sources of Na^+^, such as albite (plagioclase) weathering or ion exchange (i.e. natural softening). Ion exchange occurs when the equivalent ratio of Na^+^ versus (Na^+^ + Cl^−^) is greater than 0.5 (Hounslow, 1995). The calculated ratio for thermal water samples shows a value of 0.6, corroborating the results obtained by the (Ca^2+^  + Mg^2+^) − (HCO_3_^−^  + SO_4_^2−^) versus (Na^+^  + K^+^  − Cl^−^) ratio (Fig. 6d).

**Fig. 8:**
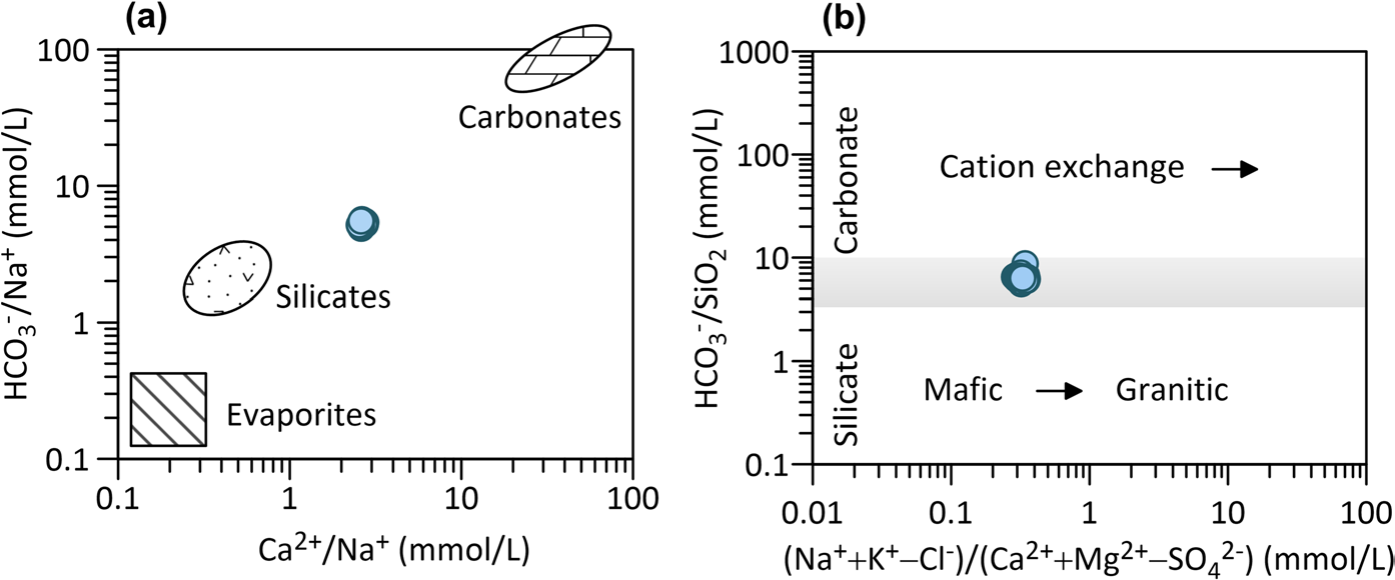
Molar ratios in thermal water samples as indicators of silicate versus carbonate weathering (**a**, modified after I. Clark, 2015; Gaillardet et al., 1999). Chloride and sulphate corrections are made to account for evaporite contributions in the right-hand chart (**b**)

Ca^2+^/Na^+^ and HCO_3_^−^/Na^+^ ion ratios can be used to distinguish between silicate and carbonate weathering (Fig. 8a).

Research by Drever and Hurcomb in (1986) (cited in Hounslow, 1995) has shown that the process of silicate weathering leads to a water Na/Ca ratio resembling that of the plagioclase mineral it originated from. The molar ratios of Ca^2+^/Na^+^ greater than 10 imply solely carbonate weathering in the catchment area. Contrarily, the weathering of feldspar produces more alkali cations, and as a result, the Ca^2+^/Na^+^ ratio is typically smaller than 1 (Clark, 2015; Gaillardet et al., 1999). The Ca^2+^/Na^+^ molar ratio of 3 in Topusko thermal water samples is a manifestation of approximately 25–30% of silicate weathering and around 75% of carbonate weathering. Figure 8b corroborates previous indications of dominant carbonate dissolution and cation exchange processes in the thermal aquifer.

**Fig. 9:**
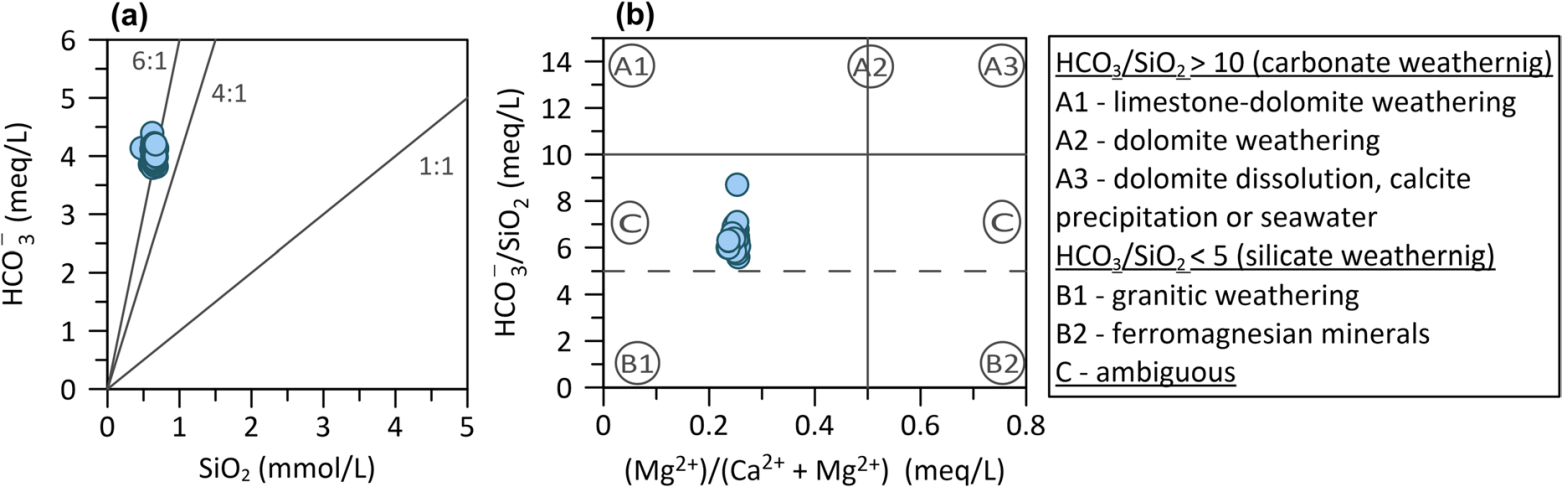
Biplot of HCO_3_^−^ vs SiO_2_ ratio and HCO_3_^−^ /SiO_2_ vs Mg2^+^/(Ca2^+^ + Mg2^+^) equivalent ratio. Modified from Hounslow (1995) and (de Carvalho Filho et al., 2022)

Considerable silica is released into solution by weathering albite and orthoclase (alkali feldspar) compared to other silicates (Clark, 2015; Hounslow, 1995). An arbitrary division of dominant silicate or carbonate weathering can be performed using the HCO_3_^−^/SiO_2_ molar ratio being less than five or greater than ten for predominant silicate or carbonate dissolution, respectively. Figure 9a displays the bicarbonate/silica ratio of around 6 in the investigated thermal water. In addition, the ratio of Mg^2+^/(Mg^2+^  + Ca^2+^) greater than 0.5 would indicate silicate weathering, whereas in Topusko thermal water samples is 0.25, implying limestone-dolomite weathering process occurring during water chemical evolution (Fig. 9b). Thermal water samples fall between categories where the interpretation of results is ambiguous. The ratio of SiO_2_/(Na + K-Cl) between values of 1 and two is indicative of albite weathering and less than 1 of cation exchange. Thermal water samples take on the value around 1.2, again corroborating the previously deducted impression on albite weathering in the aquifer or along the flow path.

If some uncertainties are still present, it is advisable to look at TDS values, which are usually lower for dominantly silicate weathering in the system (100–200 mg/L) than dominantly carbonate (500 mg/L or higher). In addition, considering the regional geological map, the surface outcrops of silicate lithologies (Jurassic siliciclastic deposits) are present at the surface SW from Topusko and can be expected in the subsurface.

The studied ratios point to carbonate dissolution as the main source of major ions and the ion exchange process in the thermal aquifer as the dominant source of Na^+^ occurrence. The ion exchange process might occur in the final part of thermal water rise to the surface due to contact with Miocene sediments and Cretaceous flysch (Mišić, 2022).

**Fig. 10:**
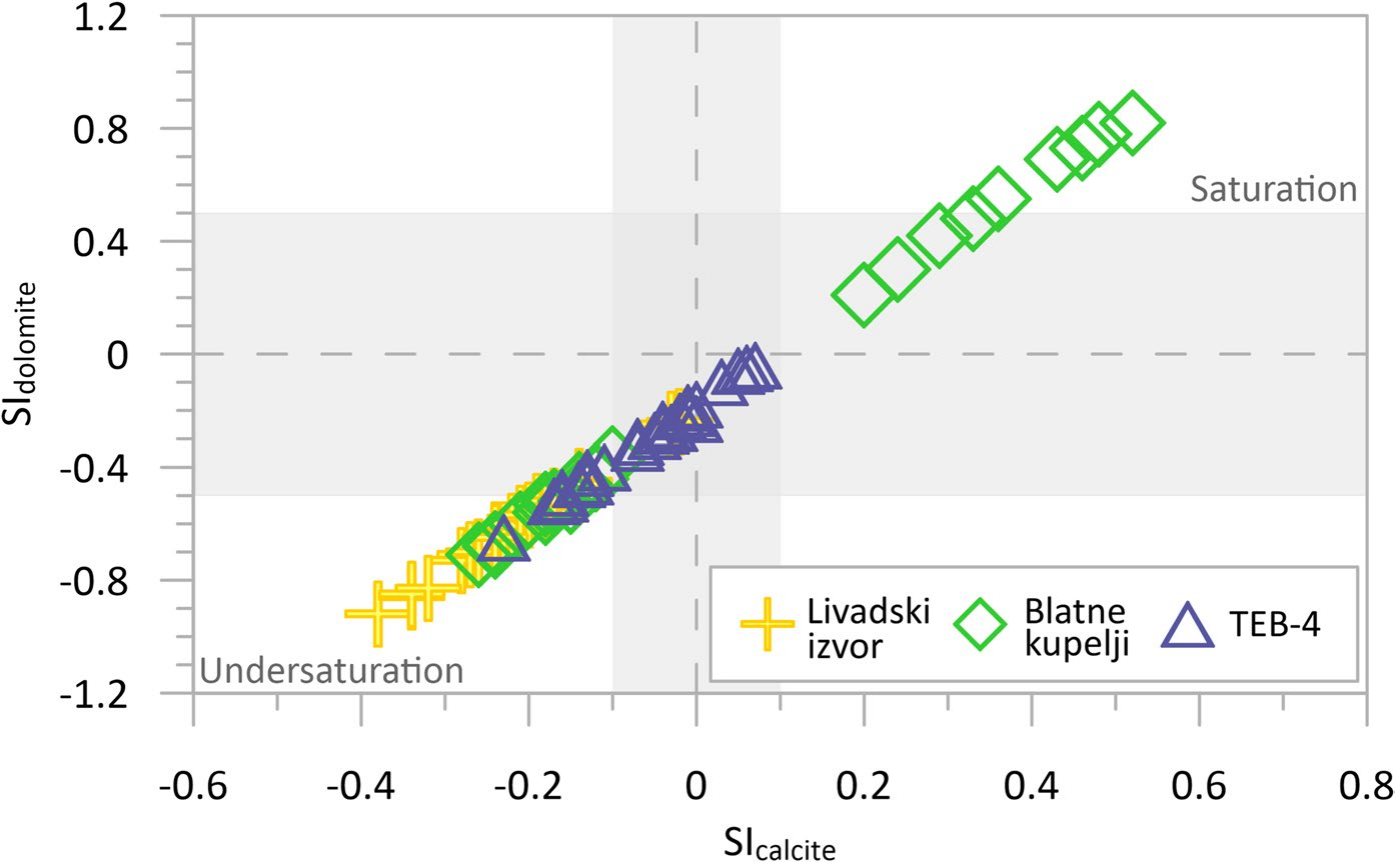
Calculated saturation indexes (SI) with respect to calcite and dolomite for three sampling objects

Since the main thermal aquifer is composed of calcite and dolomite, their SIs were further investigated (Fig. 10). The results show that most samples from TEB-4 have saturation index values close to zero, falling within the uncertainty range (grey rectangle) and indicating that calcite and dolomite are in equilibrium in these waters. On the other hand, most of the samples from Livadski izvor spring had saturation index values below zero, indicating undersaturation. These results underline the dynamic nature of the interactions between minerals and thermal water, with most samples of TEB-4 showing a state of equilibrium in terms of calcite and dolomite saturation. Such results suggest that equilibrium water–rock interaction had been attained in the aquifer (Appelo & Postma, 2005).

### Stable water isotopes

The δ^18^O and δ^2^H values for the thermal waters in the study area ranged from − 11.03‰ to − 10.73‰ and from − 76.02‰ to − 74.28‰, respectively. A total of 24 precipitation samples were analysed, with values of δ^18^O and δ^2^H ranging from − 13.80‰ to − 3.96‰ and from − 100.21‰ to − 20.73‰, respectively (Briški et al., 2023).

#### Construction of LMWL and comparison with thermal water composition

**Fig. 11:**
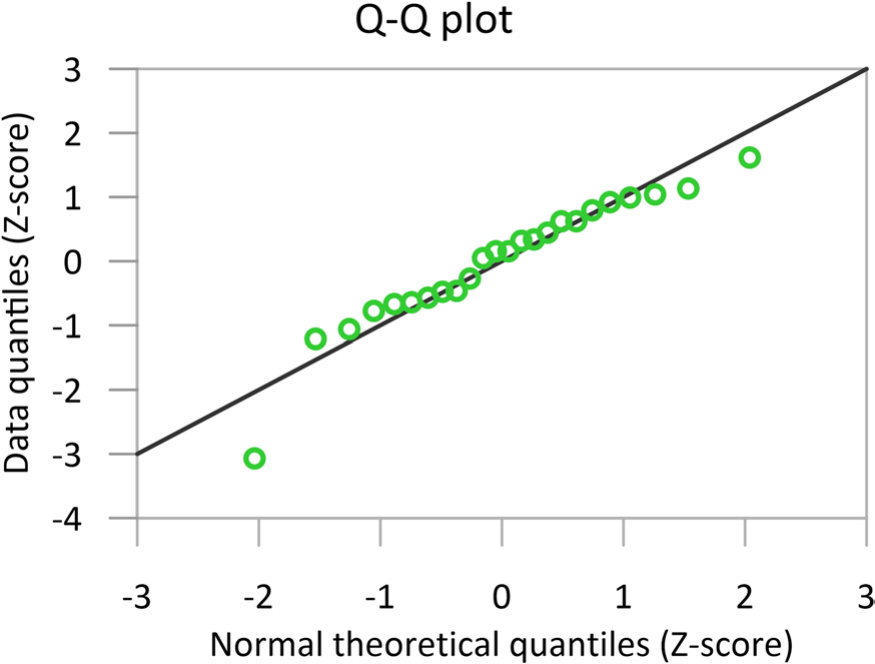
Q–Q plot of δ^2^H and δ^18^O (d-excess) precipitation data

A Q-Q plot was used to assess the normality of precipitation stable water isotope data before statistical analysis. Figure 11 indicates that most d-excess normalised values closely adhere to a theoretical normal distribution. However, one data point deviates significantly from the dataset. Despite this outlier, the W-test suggests that the remaining values do not deviate substantially from normal distribution, with an asymmetrical skewness. Using Chauvenet's Criterion, the outlier identified was removed (precipitation sampled in August 2022), and subsequent analysis showed that the dataset now exhibited a normal distribution with potentially symmetrical skewness.

Based on the simple linear regression model, the local meteoric water line (OLSR LMWL) is:

$${\updelta }^{2} {\text{H = 7}}{{.98\updelta }}^{18} {\text{O + 12}}{.48}$$

**Fig. 12:**
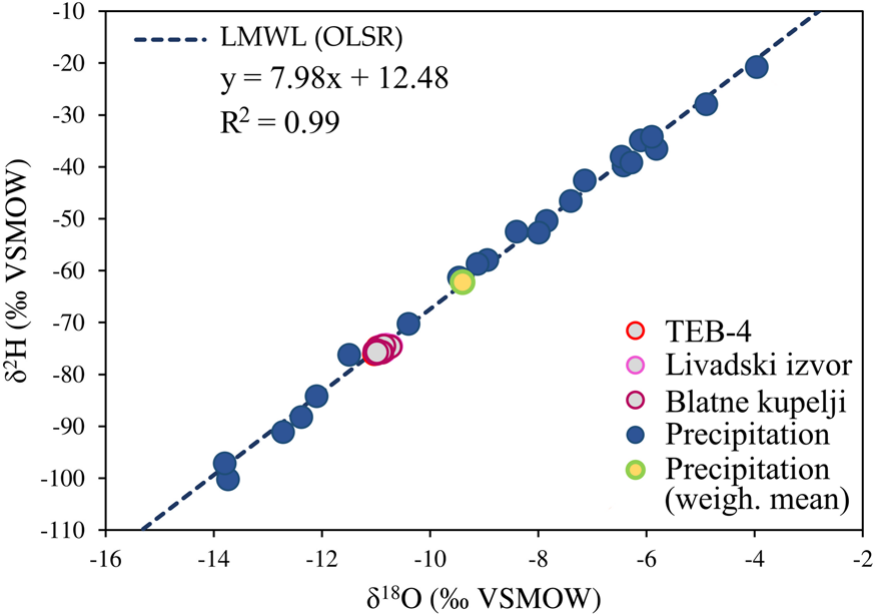
Isotopic composition δ^2^H and δ^18^O of all collected thermal water and precipitation samples in the area of the Topusko hydrothermal system together with calculated OLSR LMWL

The diagram in Fig. 12 shows the isotopic composition (δ^2^H and δ^18^O) of all collected samples of thermal waters and precipitation in the area of the Topusko hydrothermal system together with calculated OLSR LMWL. The thermal water samples are distributed on the LMWL, confirming the meteoric origin of discharged thermal water in the spring area and showing that the secondary processes, such as evaporation of precipitation before infiltration, are negligible (Mazor, 2004). The weighted arithmetic mean value, calculated following Mance (2014), of the collected precipitation samples is δ^18^O =  − 9.40 ‰ (Fig. 12). Typically, the weighted mean annual value of δ^18^O and δ^2^H in precipitation represents the isotopic signature of groundwater (Clark, 2015). The mean value of the thermal water samples is approximately − 10.92‰ with minor variations among the sampled objects. Lower mean values of stable isotopes of thermal waters in relation to weighted mean precipitation values (-1.51 ‰) may indicate a different area of recharge (i.e. a higher altitude) or that the recharge took place in colder climatic conditions in comparison with present (Bayari et al., 2009; Mazor, 2004; Porowski, 2014). Since there are no substantial changes in altitude in the investigated area (Fig. 2), the observed shift could be justified by different climatic conditions, suggesting that the Topusko thermal waters are relatively old.

#### Paleogroundwater

Paleogroundwaters, recharged during past glaciation events, exhibit distinct isotopic characteristics, being isotopically depleted relative to modern meteoric groundwaters and shifted along GMWL towards negative values (Clark et al., 2000; Grasby & Chen, 2005; Porowski, 2014). The "paleoclimatic effect" was exemplified by Porowski (2014) in the Great Hungarian Plain, where Pleistocene recharge had δ^18^O values below − 10 ‰ and the values of ^14^C activity less than 10 pMC (i.e. 20 ka BP). To account for potential altitude effects, δ^2^H and δ^18^O values from nearby measurements in Zagreb and Mt. Medvednica (10 km north of Zagreb station) were considered, revealing a consistent vertical isotopic gradient of − 0.28‰ per 100 m (Krajcar-Bronić et al., 1998; Vreča et al., 2006). Kern et al. (2020) recommend the use of values of − 1.2‰/km for δ^18^O and − 7.9‰/km for δ^2^H as an 'altitude' effect in the Adriatic Pannonian region for modern precipitation. This information allowed for the estimation of precipitation recharge elevation from ≈700 to ≈1200 m above sea level of THS. Notably, the absence of high mountains in the region supports the idea of geothermal aquifer recharge during different climate conditions, indicating paleogroundwater in Topusko.

In the assumed recharge area, d-excess values for precipitation ranged from 9.73‰ to 15.81‰, with thermal water values falling within this range, averaging 12.1‰. These observed d-excess values reflect the climate conditions at the time of recharge, which may have been notably different from the present. While the study area is close to the Mediterranean coast, it appears that continental precipitation is the primary source of recharge, as suggested by mixing of vapour sources in thermal water d-excess values (Kostrova et al., 2020; Chizhova et al., 2022).

### Tritium content

Table 2 shows tritium concentrations of analysed thermal water samples. The tritium activity concentrations for samples collected during the minimum abstraction rates are below the detection limit. The absence of detectable tritium suggests that geothermal waters are sub-modern (Motzer, 2007) and have infiltrated the subsurface before 1950.

**Table 2:** Results of tritium activity concentrations of thermal water samples

Well, sampling date	Depth (m)	Conditions	Bq/L	TU (tritium unit)
TEB-4; 12.5.2022	80.8	Maximal use of thermal water*	0.10 ± 0.10	0.89 ± 0.82
TEB-4; 13.9.2022	80.8	Minimal use of thermal water	0.02 ± 0.03**	0.14 ± 0.28**

^*^ The end of the district heating season, which lasts around 205 days. ** Below detection limit

The tritium concentration of the sample collected after the heating season, when the abstraction rates are maximal, was determined to be 0.89 ± 0.82 TU, indicating the mixing of sub-modern and modern water (Motzer, 2007). However, this interpretation should be carefully considered since the measured value is at the limit between the two categories. This result could suggest a mixing of the thermal water with modern water from shallow aquifers connected to a local pressure drop in the thermal aquifer due to thermal water abstraction for heating and health purposes. Although the thermal aquifer is confined and artesian, its upper confining layer is probably leaky, providing a connection with the shallow colder aquifer hosted in the Quaternary cover. Such results are not representative of tritium content in the thermal aquifer. Similar tritium concentration activity results in thermal springs and interpretations were reported by Young (1985).

### Carbon isotopes of dissolved inorganic carbon (DIC)

Topusko thermal water samples have ^14^C activity of 11.6–13.1 pMC and ^14^C DIC apparent age between 16,330 and 16,790 ± 40 years BP (Table 3). Conventional radiocarbon age results suggest that the groundwater was recharged during the Late Pleistocene, close to the last glacial maximum (LGM), around 18,000 years BP (Clark et al., 2009; Hughes et al., 2013, 2022a; Prell et al., 1980), by paleo-precipitation. High values for the δ^13^C measured with respect to VPDB in water samples are consistent and range from − 4.1 to − 4.3 ‰, indicating possible dilution with "dead carbon", mixing or isotopic exchange (Gallagher et al., 2000).

**Table 3:** Carbon isotope data of DIC and ^14^C apparent age of Topusko thermal water

Well, sampling date	Conditions	^14^C (pMC)	^14^C (pmc)	δ^13^C (‰ VPDB)	Apparent DIC ^14^C age (BP)
TEB-4; 12.5.2022	Maximal use of thermal water*	13.1 ± 0.1	26.8	-4.3	16 330 ± 40
TEB-4; 13.9.2022	Minimal use of thermal water	12.4 ± 0.1	25.3	-4.3	16 790 ± 40

^*^The end of the district heating season, which lasts around 205 days

#### Single-sample-based correction models

Traditional adjustment models (Eichinger, 1983; Fontes & Garnier, 1979; Ingerson & Pearson, 1964; Mook et al., 1974; Plummer & Sprinkle, 2001; Tamers, 1975) were used to calculate initial ^14^C content and adjusted ^14^C ages based on DIC content from a single well TEB-4 and major ions composition data. The parameters used in traditional models' calculations via NetpathXL are assumed to be 100 pMC for the initial ^14^C value of the soil CO_2_, solid carbonate minerals are assumed to have δ^13^C of 0 ‰ and ^14^C of 0 ‰ and the δ^13^C of soil gas CO_2_ was calculated by assuming that the dissolved CO_2_ is in isotopic equilibrium with the soil gas (Han & Plummer, 2016). However, in reality, these values exhibit spatial and temporal variations, impacting the certainty of ^14^C age estimations, which depend on both model choice and estimated ^14^C and ^13^C values of soil CO_2_ and carbonate minerals (Han & Plummer, 2016; Wood et al., 2014). Concentrations of total dissolved inorganic carbon (TDIC) were assumed to be equal to bicarbonate (alkalinity), considering the pH-dependent distribution of dissolved carbonate species (Mook, 2000). In most systems closed to soil CO_2_, HCO_3_^−^ is the predominant species, and TDIC is mainly in the form of alkalinity (HCO_3_^−^) (Bottrell et al., 2019; Han & Plummer, 2016).

#### Han graphical method

**Fig. 13:**
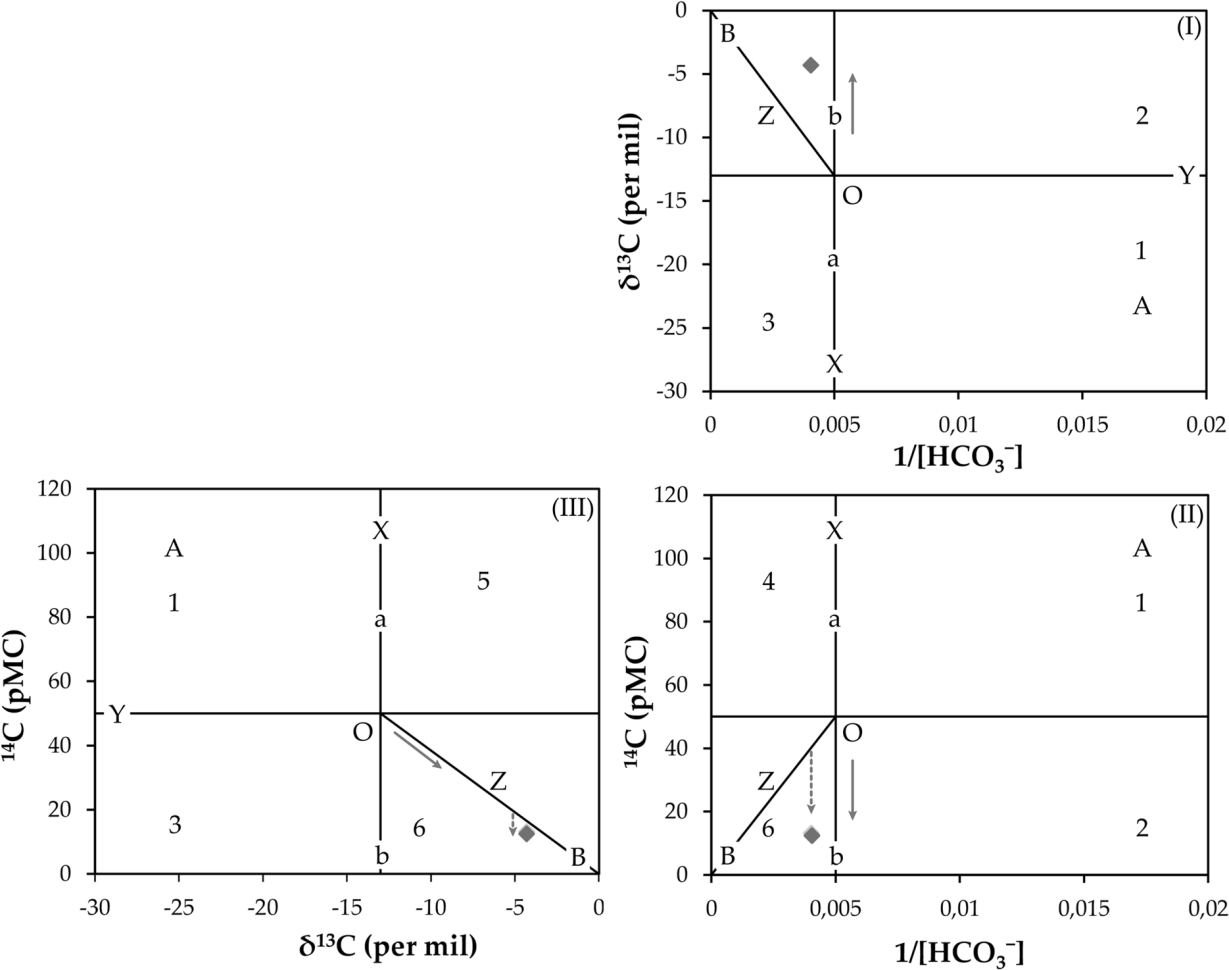
Graphical representation modified after Han et al. (2012) shows chemical and isotopic evolution of DIC in Topusko thermal water. The full-line arrows represent the isotopic exchange between water and solid carbonate, and the dashed-line arrows represent the decay of ^14^C

The Han et al. (2012) graphical method was used to identify geochemical processes that affect the chemical composition of thermal water and for qualitative estimation of the radiocarbon age of thermal groundwater samples. The graphs in Fig. 13 show the relationship between δ^13^C (‰) and the reciprocal of DIC concentration ([DIC]) (I), the relationship between measured ^14^C activity (pMC) and 1/[DIC] (II), and the relationship between measured ^14^C activity (pMC) and δ^13^C (‰) (III). It is assumed that DIC and HCO_3_^−^ concentrations are almost equal regarding Topusko water chemical evolution following recharge, which is determined by plotting data points left of line X in graphs I and II. Since the value of δ^13^C soil gas CO_2_ at the time of recharge is unknown, it is assumed to be − 26 ‰, which is today's measured average concentration of C_3_ type of plants (Cerling et al., 1993; Han & Wassenaar, 2021; Mook, 2000). The initial ^14^C value of soil CO_2_ is assumed to be 100 pMC. The assigned ^14^C and δ^13^C values for carbonate minerals are 0 pMC and 0 ‰, respectively (Han & Plummer, 2016). In our study, Tamer's point, the 'primary' carbon isotopic composition of DIC (*mainly CO*_*2*_*(*_*aq*_*)* + *HCO*_*3*_^−^*)* is located at − 13 ‰ for δ^13^C and 50 pMC. A more detailed explanation of diagram construction and application is provided by Han et al. (2012).

The samples are plotted in region 6 of the Han graph (Fig. 13; (II) and (III)), typical for "old waters". Samples plotting in this region are expected to have undergone ^14^C decay. It is observed that the data plot very close to the carbonate dissolution line, which is indicative of a strong dilution of carbon isotopic content and a downward shift point to ^14^C decay. The Han graph indicates two possible major processes influencing DIC and carbon isotope composition (^13^C and ^14^C) in thermal water: (i) isotopic exchange between water and carbonate and (ii) incongruent dissolution of carbonates. The effect of "dead carbon" introduced by the dissolution of limestones and dolomites changes the isotopic composition by increasing δ^13^C and diluting ^14^C concentrations from crossing point O, as carbonates are assumed to have ^14^C-free DIC (~ 0 pMC). It is to reiterate that if groundwaters are old, water–rock interaction might cause the loss of the initial isotopic signature (Han & Plummer, 2016). Based on the graphs in Fig. 13, the age of thermal water could be estimated to be around 9,250 years BP, from an initial ^14^C content of ca. 40 pMC (vertical intersection with zero-age line Z, on graph II).

#### Radiocarbon age of Topusko thermal water

The radiocarbon age calculated by application of traditional models (Plummer & Glynn, 2013) and Han et al. (2012) graphical method is presented in Table 4.

**Table 4:** The corrected initial activities of ^14^C DIC values and calculated radiocarbon ages using traditional adjustment models calculated via NetpathXL and Han et al. (2012) graphical method

Well	Uncorrected age	Selected adjustment models											
		Mass balance		Tamers		Ingerson and Pearson		Mook		Fontes and Garnier		Han graph	
	Age	A_0_	Age	A_0_	Age	A_0_	Age	A_0_	Age	A_0_	Age	A_0_	Age
	BP	pmc	BP	pmc	BP	pmc	BP	pmc	BP	pmc	BP	pmc	BP
TEB-4 12.5.2023	16,330	60.04	6,668	67.26	7,607	68.81	7,795	91.95	10,192	71.88	8,155	40.20	9,010
TEB-4 13.9.2023.*	16,790	59.56	7,078	67.69	8,136	68.26	8,205	92.16	10,687	69.51	8,355	40.40	9,491

^*^NetpathXL programme uses modern ^14^C half-life (5730) and needs to be converted to Libby half-life (5570) using the equation tlibby = 0.972t_5730_ (L. N. Plummer & Glynn, 2013). The age differences range from 187 years to 299 for the oldest age estimation, and negligible differences can be assumed for this study and method

The relationship between ^3^H and ^14^C content in groundwater and ^13^C and ^14^C in nature, schematically presented by Mook (2000), indicates the presence of old groundwater in the Topusko aquifer, which has a carbon isotopic footprint more similar to fossil carbonates than aged groundwater. The identified geochemical processes, which account for the reservoir effect and result in δ^13^C enrichment and ^14^C depletion, are strong and backing up the use of de-normalised ^14^C DIC values ('pmc') for calculation of radiocarbon age by application of traditional adjustment models to a single water analysis in the system. The various models generated corrected values of initial ^14^C (A_0_) content ranging from 59 to 100 pmc, which gave thermal water residence times of 6,668 to 10,687 years BP, based on the ^14^C activity measured in thermal water samples. The average residence time obtained from traditional models is 8,448 years BP and 9,250 years BP for qualitative estimation using the Han–Plummer plot (A_0_ ca. 40 pMC). Horvatinčić et al. (2012) reported groundwater age for a similar ^14^C activity value from the Zagreb dolomite geothermal aquifer at 11,650 ± 620 years BP but without correction for the reservoir effect.

Along with numerous empirical methods that have been used to estimate A_0_, Geyh (2000) calculated a set of A_0_ values that would be better suited for characterising the initial ^14^C activity of DIC in water discharging from distinct aquifer geological settings, which were proven to exhibit strong agreement with A_0_ values frequently derived more rigorously through independent modelling. In the case of THS, where the catchment area is dominantly built of carbonates, thermal water age corrections of − 3,500 to − 5,000 can be expected, with the estimated initial ^14^C activities ranging from 55 to 65 (pMC). Such calculations can roughly be considered in accordance with the results obtained for THS with the traditional model of the Mook and Han graphical method (Table 4).

Within the context of the geological time scale, the recharge of the Topusko geothermal aquifer might have occurred during the Late Pleistocene and the beginning of the Holocene, coinciding with the end of the Last Glacial Cycle—a global ice expansion period (Goñi, 2022; Palacios et al., 2022). During this period, most of western and central Europe and Eurasia was open steppe-tundra, while the Alps presented solid ice fields and montane glaciers (Li, 2022). Research by Hughes et al. (2022b) suggests that some of the lowest Pleistocene glaciers in Southern Europe formed in the coastal Dinaric Alps bordering the Adriatic Sea. During the LGM (Hughes et al., 2013), average global temperatures were around 8.3 ± 1.5 °C, with year-round ice covering about 8% of Earth's surface and 25% of the land area, while currently (as of 2012) about 3.1% of Earth's surface and 10.7%, respectively (Dubey, 2023). This suggests that climate conditions during the recharge were colder than present, corroborating the stable water isotope signature.

### Stable sulphate anion isotopes δ^34^S and δ^18^O of SO_4_^2−^

The results of δ^34^S and δ^18^O of SO_4_^2−^ anion in thermal water samples are presented in Table 5. The analysis found a consistent isotopic composition across sampled thermal waters, with average values of 9.3 ‰ and 8.35 ‰ for δ^34^S and δ^18^O, respectively. This uniformity indicates a stable source or process governing the sulphate composition within this hydrothermal system.

**Table 5:** δ^34^S and δ^18^O isotopic composition of SO_4_^2−^ anion in thermal water samples

Well, sampling date	Conditions	δ^34^S (‰) (CDT)	δ^18^O (‰) (VSMOW)
TEB-4; 12.5.2022	Maximal use of thermal water*	9.2	9.3
TEB-4; 13.9.2022	Minimal use of thermal water	9.4	7.4

^*^The end of the district heating season, which lasts around 205 days

**Fig. 14:**
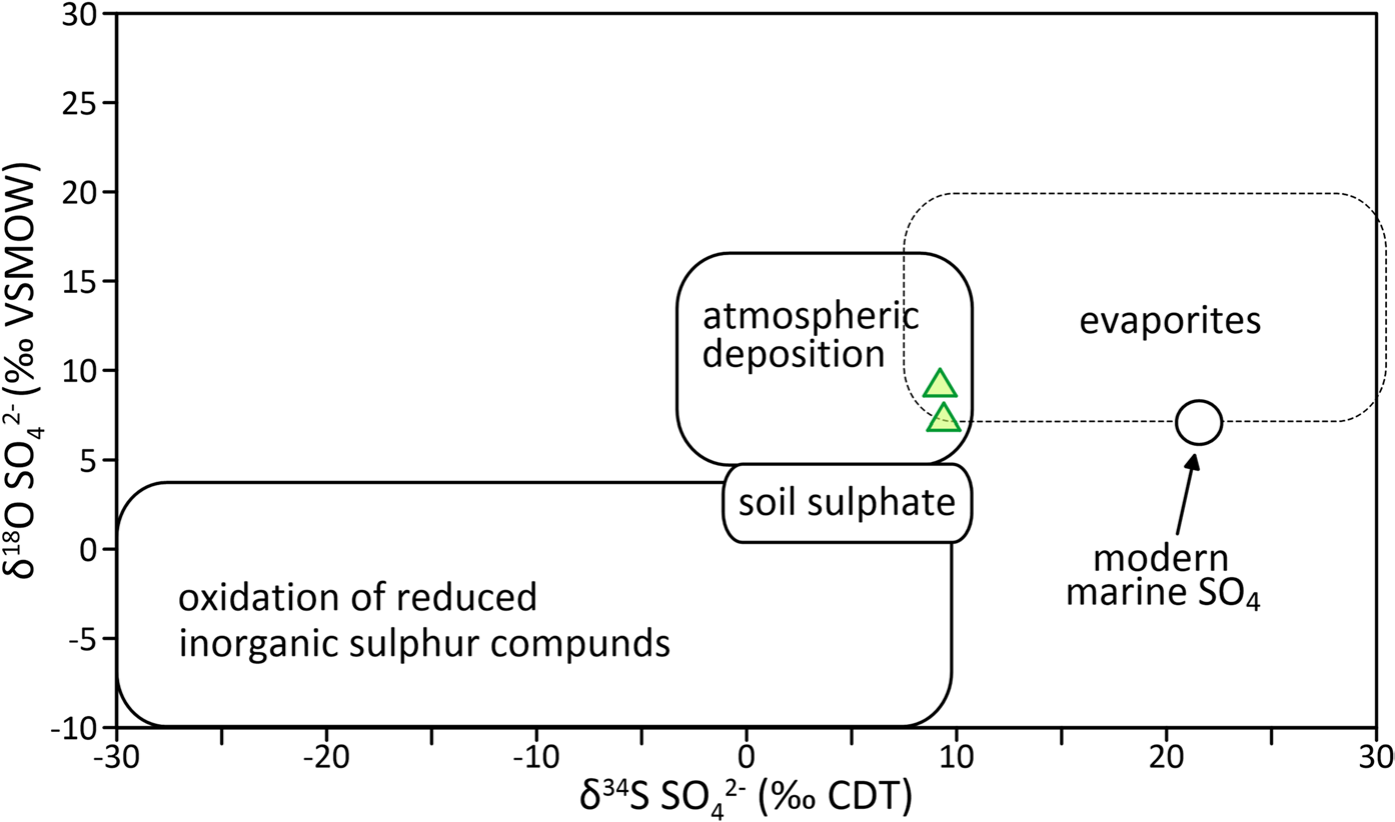
The δ^34^S versus δ^18^O of dissolved SO_4_^2−^ in thermal water of Topusko aquifer (triangles) versus the background values of typical sulphate sources (after Porowski et al., 2019)

According to the ratio of δ^34^S and δ^18^O of SO_4_^2−^, the sulphate in the Topusko water could be of atmospheric or evaporitic origin (Fig. 14; Porowski et al., 2019).

The atmospheric deposition (i.e. rain and snowmelt) as a source of the sulphates in the Topusko water can be excluded since the atmospheric input usually results in low concertation of sulphates. Conversely, the Topusko water has a relatively high concentration of sulphates (Table 1), which is higher than other thermal and fresh waters in Central Croatia (Borović, 2015; Marković et al., 2015; Nakić et al., 2013). Furthermore, it is unlikely that the isotopic signature of sulphates reflects the current atmospheric conditions due to the long residence time of the thermal water suggested by δ^18^O, δ^2^H, ^3^H, and ^14^C.

The presence of sulphate in the Topusko thermal water may be linked to the dissolution of evaporite sulphates (gypsum and/or anhydrite). The enrichment of sulphates with heavier isotopes, such as oxygen ^18^O and sulphur ^34^S, is a characteristic sign of evaporite sulphate dissolution. Gypsum is known to accumulate in the soil of arid regions and often occurs alongside dolomite and limestone. Given the geological composition of the Topusko geothermal aquifer, which predominantly consists of carbonates, the presence of evaporites below the aquifer is plausible. Geological mapping of the region identified gypsum outcrops NW of Cetingrad (≈ 20 km SW from Topusko), likely part of an Upper Permian evaporite sequence associated with fibrous gypsum and fine-grained primary dolomite (Korolija et al., 1980b).

Gypsum and anhydrite dissolve without isotope fractionation, which enables direct use of the isotopic composition of SO4_2_^−^ as a tracer for the sulphate origin (Porowski, 2014). By comparing the sulphate isotope content in Topusko thermal water to global measurements of marine evaporite deposits, notable variations in δ^34^S values over geological time were observed. These values ranged from around + 35 ‰ during the Cambrian to less than + 10 ‰ in the Permian period. Similarly, δ^18^O values fluctuated from approximately + 20 ‰ to around + 7 ‰ (Claypool et al., 1980). Previous studies, such as Forzis et al. (2019) in Budapest (Hungary), have used similar analyses to determine the origin of dissolved sulphates in thermal waters and concluded that the dissolved sulphate in thermal water is mainly a product of Permian evaporite dissolution. It is possible to assume that the presence of dissolved sulphate in Topusko thermal water, characterised by an average δ^34^S value of 9.3 ‰ and an average δ^18^O value of 8.35 ‰, can be attributed to evaporite dissolution.

### Geothermometers

The equilibrium thermal water temperature in the thermal aquifer was estimated by several classical chemical geothermometers (Table 6).

**Table 6:** Temperatures (°C) calculated using experimentally calibrated chemical geothermometers (silica and cationic) for the Topusko thermal water samples

Sampling location	a (°C)	b (°C)	c (°C)	d (°C)	e (°C)	f (°C)	g (°C)	h (°C)	i (°C)	j (°C)	k (°C)	l (°C)	m (°C)
Livadski izvor spring	89.7	89.5	90.7	58.8	60.8	90.3	525.7	360.83	388.0	415.7	264.4	117.7	63.9
Blatne kupelji spring	89.3	89.2	90.4	58.4	60.5	89.9	526.1	360.99	388.2	415.8	262.5	118.0	64.0
TEB-4 well	90.8	90.6	91.8	59.9	61.9	91.4	526.5	361.21	388.6	416.1	264.4	116.9	64.0
Average	89.9	89.8	91.0	59.0	61.1	90.5	526.1	361.10	388.3	415.8	263.8	117.5	64.0

(a—Truesdell, 1976), (b—Fournier, 1977), (c—Michard, 1979), (d—Verma & Santoyo, 1997), (e—Fournier, 1977), (f—Arnórsson et al., 1983), (g—Michard, 1979), (h—Fournier, 1977), (i—Truesdell, 1976), (j—Fournier & Truesdell, 1973), (k—Benjamin et al., 1983), (l—(Chiodini et al., 1995), (m—Giggenbach, 1988))

The Na–K (g, h, i; Table 6) and Na–K-Ca (j, k; Table 6) geothermometers provide an average equilibrium temperature of thermal water from 263.8 °C to 526.1 °C, which is not realistic, based on the geological and hydrogeological setting in the study area, and therefore are rejected. Furthermore, these geothermometers are generally considered inadequate for low-temperature reservoirs/aquifers (Karingithi, 1984). The Ca-Mg geothermometer (l; Table 6) yields elevated temperature values, approximately 117 °C, which can potentially be influenced by uncertainties due to the disorder degree of dolomite (Blasco et al., 2017; Bruno et al., 2020). The values obtained by K-Mg geothermometers were 64 °C, similar to those obtained by SiO_2_-chalcedony (approximately 60 °C), close to the wellhead and spring temperature values. These calculations are unrealistic because the temperature of the water can decrease slightly during its ascent to the surface, and there is no mechanism which would cause the water to heat up during outflow. The calculated average aquifer equilibration temperature by quartz geothermometers (a,b,c; Table 6) is 90 °C. Pavić et al. (2023) reported the predicted average aquifer temperature of THS to be approximately 78 °C using quartz geothermometers. The discrepancy could be related to the lower silica concentrations in the historical hydrochemical data. Despite the small difference, both results could be considered realistic since quartz geothermometers are generally considered the more accurate for low-temperature systems with waters with near neutral pH (Blasco et al., 2018, 2019; Borović, 2015; Rman, 2009; Witcher & Stone, 1983).

## Conclusions

The paper presented and discussed the results of the first systematic monitoring of the thermal waters of the THS, and the main conclusions which can be drawn therefrom are as follows:

1. Principal ion chemistry data show that the Topusko thermal waters display Ca-HCO_3_ hydrochemical facies, confirming the influence of geological formations dominated by carbonate rocks. In THS, around 75% of carbonate weathering and 25–30% of silicate weathering occur. High concentrations of Ca^2+^, Na^+^ and Mg^2+^ were caused by mineral dissolution and cation exchange. Considering the available historical data, thermal water composition is stable. The differences were observed in the higher silica content at present, possibly due to different measurement methodologies. Ion exchange and silicate weathering cause the increase of Na^+^ from the recharge to the discharge zone.
2. Stable water isotope data, δ^2^H and δ^18^O, suggest that the recharge of thermal water is of meteoric origin, i.e. precipitation. Lower mean values of stable isotopes (-1.51 ‰ in δ^18^O) of thermal waters in relation to weighted mean precipitation values indicate that the recharge took place in colder climatic conditions compared to the present. The δ^18^O values of thermal water are very uniform, from − 11.3 ‰ to − 10.73 ‰, indicating deep circulation and large areal extent and thickness of the aquifer, with longer residence times in which seasonal variations of precipitation are homogenised.
3. Additionally, ^14^C dating of DIC shows that thermal water has residence time ranging from 6,668 years BP to 10,687 years BP. The average residence time obtained from traditional models is 8,473 years BP and 9,536 years BP for qualitative estimation using the Han–Plummer plot. These ages also suggest that thermal water is representative of the colder climate in the late Pleistocene or early Holocene.
4. Tritium activity in thermal water is below the detection limit. However, after the period of extensive abstraction for district heating during winter, some tritium activity (around the detection limit) was measured in thermal water samples. Possible infiltration of modern precipitation from younger layers above is possible, as the hanging wall is not absolutely impermeable. Regular and precise measurement of tritium activity would be very useful for understanding if the abstraction rates are sustainable.
5. Chemical geothermometers were used to estimate the maximum equilibrium temperature reached by thermal waters in the aquifer. The quartz geothermometer provided the most plausible equilibrium aquifer temperature of 90 °C.
6. Data on stable sulphate anion isotopes, δ^34^S and δ^18^O, point to gypsum and/or anhydrite dissolution at depth. Deeper boreholes or seismic profiles do not exist, so this assumption is based solely on hydrochemical data.

All of the mentioned analyses and interpretations give valuable information for the development of the conceptual model of THS by constraining the hydrogeochemical processes that drive the solute content, determining the source of recharge of the hydrothermal system, and thermal water mean residence time. Also, this research provides a quality baseline for the future monitoring and management activities of Topusko hydrothermal system, which is highly recommended due to the continuous utilisation of the resource.
